# On the underappreciated role of scavengers in freshwater ecosystems

**DOI:** 10.1093/biosci/biaf032

**Published:** 2025-04-12

**Authors:** Morgan L Piczak, Robert J Lennox, Knut W Vollset, Bálint Preiszner, Tibor Erős, Grégory Bulté, Matt G Keevil, John S Richardson, Steven J Cooke

**Affiliations:** Department of Biology, Institute of Environmental and Interdisciplinary Science, Carleton University, Ottawa, Ontario, Canada; Department of Biology, Dalhousie University, Halifax, Nova Scotia, Canada; Department of Biology, Dalhousie University, Halifax, Nova Scotia, Canada; NORCE Norwegian Research Centre, Laboratory for Freshwater Ecology and Inland Fisheries, Bergen, Norway; HUN-REN Balaton Limnological Research Institute, Fish and Conservation Ecology Research Group, Tihany, Hungary; HUN-REN Balaton Limnological Research Institute, Fish and Conservation Ecology Research Group, Tihany, Hungary; Department of Biology, Institute of Environmental and Interdisciplinary Science, Carleton University, Ottawa, Ontario, Canada; School of Natural Sciences, Laurentian University, Sudbury, Ontario, Canada; Department of Forest and Conservation Sciences, University of British Columbia, Vancouver, British Columbia, Canada; Department of Biology, Institute of Environmental and Interdisciplinary Science, Carleton University, Ottawa, Ontario, Canada

**Keywords:** aquatic ecosystems, carrion ecology, aquatic subsidy, scavenging

## Abstract

The role of scavengers is well understood in terrestrial and marine systems but less so in freshwater ecosystems. We synthesized existing knowledge of scavenger ecology in freshwater, particularly within the context of the Anthropocene, including the patchy distribution of carrion, consumer responses, competition, and transfer of energy, nutrients, and diseases. We also explored ecosystem services provided by freshwater scavengers, such as direct material benefits and improvements in water quality. In addition, we examined how human activities—such as climate change, disturbance, exploitation, and fragmentation—are affecting scavenger behavior and abundance. To mitigate these anthropogenic impacts, we identified management options for environmental practitioners and decision-makers, emphasizing the importance of integrating freshwater scavenger roles into management plans and providing adequate policy protections. Finally, we highlighted key knowledge gaps, particularly regarding how changes in scavenger populations and their food sources may alter ecosystem structure and function.

Scavengers are animals that feed on the remains of animals (i.e., carrion) that died by means other than predation (e.g., road kill, disease outbreaks, senescence, insects falling in water) or by predation from another animal, therefore representing donor-controlled subsidies (Schalcher et al. 2013). Scavengers can be entirely carnivorous but are often omnivorous. Obligate scavengers obtain virtually all of their nutrition via this type of foraging, whereas facultative scavengers may also be herbivores, predators, and omnivores (DeVault et al. 2003). Facultative scavenging is more common, so that modality may be more ecologically important than previously thought (Benbow et al. 2015, Preiszner et al. 2020). As children, we learn about the important roles of scavengers in terrestrial and marine ecosystems with examples such as hyenas, vultures, and abyssal scavengers feeding on whale falls; however, scavengers also play key roles in other ecosystems such as freshwater lakes, rivers, and wetlands (including adjacent riparian areas).

Freshwater scavengers occur around the globe and include diverse taxa: fishes, amphibians, reptiles, mammals, birds, fungi, bacteria, and various invertebrates (e.g., insects, crustaceans; figure 1; Benbow et al. 2020). However, given that most freshwater scavenging occurs largely out of sight (i.e., underwater, often in turbid or turbulent waters), it tends to be less studied (Orihuela-Torres et al. 2024; e.g., there are classic reviews on scavengers in terrestrial and marine systems; see Beasley et al. 2015 and Britton and Morton 1994, respectively) and is much less likely to appear as exemplars in media. A recent literature review revealed only 206 studies that have examined carrion ecology within aquatic ecosystems (Orihuela-Torres et al. 2024). Another study determined that there was a bias toward terrestrial scavengers, with less than 20% of studies examining aquatic species (Di Marco et al. 2017). In broad syntheses exploring scavengers and the ecosystem services they provide (see O'Bryan et al. 2018, Aguilera-Alcalá et al. 2020), freshwater scavengers are barely mentioned. In short, we submit that freshwater scavengers are underappreciated and that there is need for more research on these fascinating and essential organisms.

**Figure 1.**
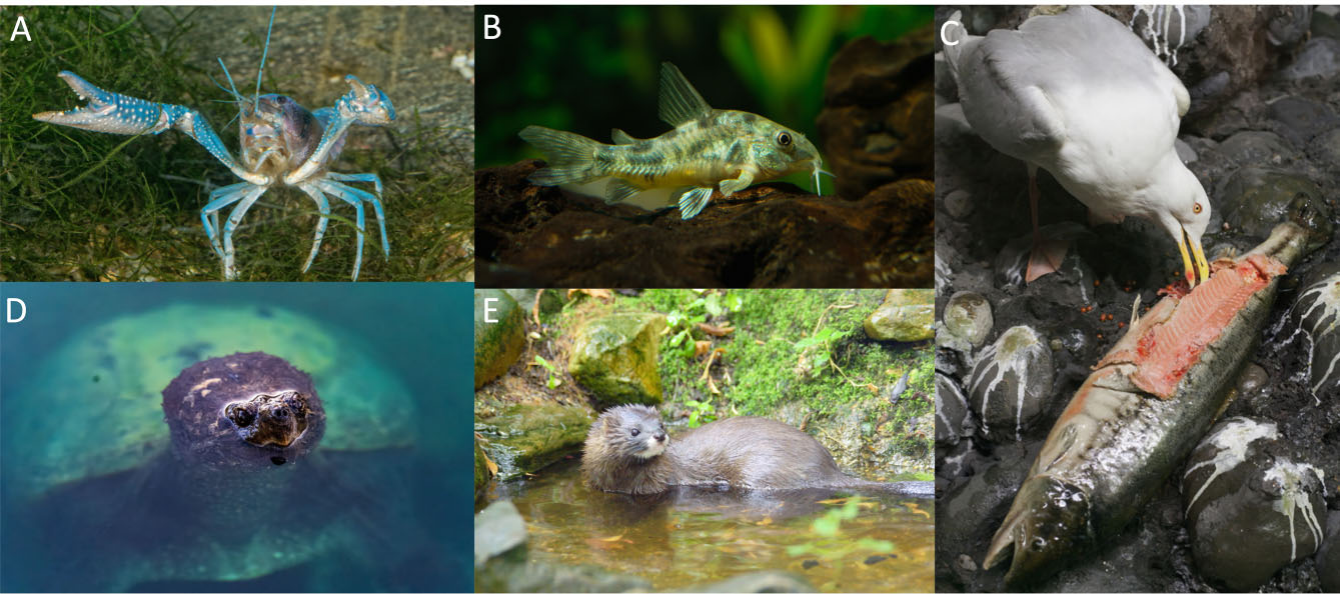
Freshwater scavengers are a diverse group across many taxa and habitat types: (a) Common yabby (*Cherax destructor*) feed on carrion in swamps, streams, and rivers within Australia. (b) Corydoras catfish (*Corydoras melanotaenia*) occur in lotic habitats throughout South America, (c) herring gull (*Larus* *smithsonianus*) inhibit many aquatic habitats throughout North America, (d) snapping turtles (*Chelydra serpentina*) are located throughout North America in slow moving waters including wetlands and ponds, and (e) European mink (*Mustela lutreola*) are found in lotic and lentic habitats.

Freshwater ecosystems are among the most threatened ecosystems on the planet (Albert et al. 2021), and it is widely accepted that we are in the middle of a freshwater biodiversity crisis (Harrison et al. 2018). Therefore, understanding how different threats may influence scavengers (or the presence and distribution of carrion) is highly relevant to environmental practitioners and decision-makers. Our goal is therefore to explore the human impacts on the scavenging ecology within freshwater systems and identify opportunities for mitigation. Building on other studies (e.g., Benbow et al. 2020 and Orihuela-Torres et al. 2024), we synthesize the ecology of scavengers in freshwater systems by considering resource pulses, consumer responses, competition, and transfer of energy. Then, we summarize the ways in which humans benefit from freshwater scavengers across multiple types of ecosystem services. Next, we consider how humans modify scavenger ecology and identify opportunities to mitigate those issues through management. Finally, we identify knowledge gaps and research needs, recognizing that freshwater scavengers are poorly represented in the literature. Notwithstanding knowledge gaps (and the fact that we limited our literature search to English), we use diverse examples from around the globe with an international team that study scavengers, mostly in freshwater and riparian systems. Although we have chosen to focus on scavengers, we acknowledge the importance of detritivores within freshwater ecosystems (see box 1).

Box 1. **The importance of detritivores within freshwater ecosystems.**

There has been much research on decomposition of plant litter in freshwaters, but considerably less on the consequences of the amount or quality of detritus on detritivore populations (e.g., Richardson and Neill 1991). Many detritivores that primarily consume dead plant material (leaves, wood) and their biofilms, are also able to scavenge on carrion given the opportunity, that is, facultative scavengers, and there is considerable evidence of invertebrates feeding directly on carcasses (Minakawa et al. 2002, Zhang et al. 2003, Fenoglio et al. 2010). This indicates that consumption of carrion and detrital materials and their biofilms is a spectrum of strategies used to take advantage of high-quality resources of carcasses that are patchy in space and time (e.g., Kohler et al. 2008). For example, studies have demonstrated higher densities of detritivores in streams with carrion, but that can be due to the fertilizing effect of nutrients from decomposing carcasses. However, few studies have directly demonstrated increased growth rate of detritivores given access to carrion. There is also evidence from increased body burden of N15 and C13, suggesting direct consumption of carrion (Kiffney et al. 2018, Kurasawa et al. 2023), but there is more direct evidence based on fatty acid profiles of normally detritivorous invertebrates consuming fish carcasses (Heintz et al. 2010, Samways et al. 2017). The most direct evidence comes from experimental studies demonstrating that some detritivorous aquatic insects directly consume carrion and have higher growth rates than on leaf litter (Chaloner and Wipfli 2002, Minakawa et al. 2002). The relative contribution to the population dynamics of such facultative scavengers remains an important research question, but higher densities of detritivores in streams with fish carcasses suggest hypotheses that functional and numerical increases do result from scavenging in addition to the consumption of vegetative materials.

## Overview of scavenger ecology in freshwater systems

Within freshwater ecosystems, there are many factors that influence the availability of dead organisms and material (summarized in table 1). First, because carrion is patchy across space and time, it needs to be detected to be used by scavengers. Next, we examine the suite of morphological, physiological, and behavioral adaptations that organisms have to be effective scavengers. Third, because the availability of carrion is inherently dynamic and, at times, quite rare, competition between scavengers can arise. Finally, all of these factors ultimately influence the transfer of energy, nutrients, and contaminants within freshwater ecosystems. In the present article, we synthesize each of these mechanisms regarding the ecology of scavengers.

**Table 1.** Summary of scavenger ecology in freshwater systems with examples.

Factor	Aspect	Description	Freshwater example	References
Patchy resource in space and time	Duration	How long the resource is available	Short-lived presence of Pacific salmon carcasses during fall spawning migrations	Schindler et al. 2013
	Magnitude	Amount of resource	Mass emergence of *Hexagenia* mayflies	Stephanian et al. 2020
	Predictability	Knowledge and reliance on availability	Alewife spawning migrations during the spring	Walters et al. 2009
Consumer responses	Morphology	Physical traits that enable scavengers to efficiently locate, consume, and digest dead organic matter	Sternal keel on giant water scavenger beetles	Matsushima 2021
	Physiological	Internal biological processes and functions that enable these animals to effectively use and process carrion	Bullhead detection of carrion resource via chemosensory ability	Preiszner et al. 2024
	Behavioral	Specific actions and patterns of activity that enable animals to locate, acquire, and efficiently carrion	Shift in gull distribution to match Pacific salmon spawning runs	Schindler et al. 2013
Competition	Resource	Aspects of the resource itself such as duration, magnitude, and predictability	Increased availability of eggs resulted in more diverse fish juvenile scavengers	Bailey and Moore 2020
	Environment	Different factors of the environment could affect accessibility and detectability and ultimately competition	Movement of carrion (e.g., dead Pacific salmonids) by water currents down a lotic ecosystem	Strobel et al. 2008
	Competitors	Intra- and interspecific, as well as interkingdom competition of carrion resources	Microbes are the first to consume the resource, with subsequent competition across kingdoms	Beasley et al. 2015
Transfer	Energy	Movement of caloric resources between ecosystem types via scavengers	Transfer from freshwater to terrestrial ecosystems through the consumption of insects	Baxter et al. 2005
	Nutrients	Movement of macro and micro nutrients between ecosystem types via scavengers	Transfer of nutrients from wildebeest carcasses into river via insects, fish, and crocodiles	Subalusky et al. 2017
	Disease	Transmission and suppression of diseases	Insects and crustaceans reduce the transmission of Ranavirus from dead, infected salamanders	Le Sage et al. 2019

### Carrion as a patchy resource

Carrion typically presents as a spatially and temporally patchy resource, representing pulses of energy and nutrients for scavengers. Reviewed in Benbow and colleagues (2020), there are many different ways that carrion can become available in freshwater ecosystems including from autochthonous or allochthonous sources from natural senescence, disease related mortalities, phenology-based deaths (e.g., mortality of Alewife, *Alosa pseudoharengus*, during annual spawning migrations in eastern North America; Walters et al. 2009, Brown et al. 2024), or stochastic deaths (e.g., mass fish die-offs; Parmenter and Lamarra 1991). Several components of the dynamics of resource supply affect how scavenger populations might respond, such as duration of availability (in relation to consumer life cycle or annual cycle), magnitude relative to annual average (biomass), and predictability (Holt 2008, Yang et al. 2008). First, carrion has a short-lived presence within most ecosystems, including freshwater and these ephemeral resources are finite and stochastic in nature (Butterworth et al. 2023), contributing to heterogeneity of landscapes (Hyndes et al. 2022). As Holt (2008) pointed out, some resources are sufficiently short-lived (and even the organisms themselves; e.g., macroinvertebrates), such that even if there is a very large pulse (e.g., Pacific salmon), it may not be possible to be an obligate consumer on such resources. On the other hand, resources that are available for a longer duration, regardless of the magnitude of the pulse, could lead to the development of obligate consumers. Even for longer duration resources, longer-lived consumers need to be able to detect those resources and subsequently move between resource patches. Next, magnitude refers to the resource availability in terms of biomass and can be influenced by cause of death, carcass location, and weather conditions (Yang et al. 2008). For example, periodic mass emergence of *Hexagenia* mayflies can inject massive amounts of biomass into freshwater ecosystems after laying eggs (Stephanian et al. 2020). Finally, predictability can range from large annual pulses occurring consistently in the same location and time—such as Pacific salmon spawners returning to their natal spawning grounds within a narrowly defined window each year (Schindler et al. 2003)—to more transient and unpredictable events, such as the occasional presence of dead animals (both vertebrates and invertebrates; Barton et al. 2013). This predictability can even drive seasonal movements or reproductive events of freshwater scavengers: that align with the timing of resource pulse availability (Levi et al. 2020).

### Consumer responses

Consumers that regularly scavenge benefit from a set of morphological, physiological, and behavioral adaptations. In terms of morphology, there are typically fewer constraints on body shape for consumers than for predators, allowing dynamic swimming performance or elevated levels of endurance because of the unpredictable availability of food (Collins et al. 2005). For example, giant water scavenger beetles have a sternal keel that extends posteriorly, which permits the beetles to stay submerged for longer with increased swimming speeds, which may aid in scavenging (Matsushima 2021). In addition, heightened sensory abilities are also favored to detect patchy, ephemeral food sources over long distances (Kane et al. 2017). Within aquatic environments, the movements of carcasses will not only be affected by water (i.e., waves) but also by buoyancy and decomposition processes, which can all affect detectability (Moleon et al. 2019). Furthermore, the most likely candidate for finding carcasses are those that have physiological adaptations, such as chemosensory apparatus, which are documented as means of food source detection in several marine scavengers (Wilson and Smith 1984, Tamburri and Barry 1999, Lisney et al. 2018). Although recent evidence suggests that chemosensory abilities may facilitate facultative scavenging (Preiszner et al. 2024) in a freshwater species, scavenging is also known in species that use primarily visual orientation (Chidami and Amyot 2008, Polačik et al. 2015). Research on the sensory apparatus used by freshwater species for carcass location is encouraged to better understand the ecoevolutionary consequences of scavenging in freshwater systems. Finally, an example of a behavioral adaptation to locate patchy food sources is the incorporation of exploratory movement patterns in the foraging behavioral toolbox. In a recent study, black bullhead (*Ameiurus melas*) was shown to use exploratory behaviors to locate carrion within complex habitats (Preiszner et al. 2024). Furthermore, species that can move rapidly across large distances are more likely to be obligate (or predominant) scavengers, whereas those with more limited dispersal capacity are likely to be opportunistic (facultative; Momot et al. 1978). Much of the existing research is focused on gregarious foraging on carcasses in terrestrial, marine, and riparian ecosystems (Clua et al. 2013, Levi et al. 2020), whereas there is a lack of evidence of similar spatial aggregations underwater in freshwater systems, with the exception of Chidami and Amyot (2008).

### Competition for carrion resources

The landscape of competition across scavengers is affected by factors associated with the resource itself, as well as the environment and competitors. Although the quantity, quality, timing, and duration framework (i.e., QQTD; Subalusky and Post 2019), describes how abiotic characteristics and aspects of the animal subsidy was not specifically developed for scavenging, there are many parallels. First, the size and type of resource (i.e., quantity and quality) can influence competition. Larger carcasses, mass availability or high nutritional content can sustain increased numbers of scavengers, reducing immediate competition (see Nowlin et al. 2007). For example, the experimental addition of large quantities of pink salmon eggs as a food resource to streams was required to maintain diverse consumers (e.g., juvenile steelhead trout, coho salmon, sculpins, cutthroat trout, and Dolly Varden char; Bailey and Moore 2020). The state of decomposition (which is also related to quality) also affects competition, in that microbes are often the first to consume the resource, with eventual competition across kingdoms (i.e., interkingdom competition) with the subsequent detection by invertebrates and vertebrates (Beasley et al. 2015). Within the environment, the accessibility of the carrion and habitat features can affect detectability (Beasley et al. 2015). For example, in terrestrial ecosystems, the presence of vegetation structure and cover can influence the detectability of carrion across soaring scavengers relative to those on the ground (Moleon et al. 2019). Additional research within the realm of freshwater ecosystems is needed where different factors of the environment would likely affect accessibility, detectability and ultimately competition. Such factors could include water quality, habitat structure (e.g., rocky or woody substrate), bathymetry, and hydrological aspects (see Preiszner et al. 2024). The scavengers themselves also affect the dynamics of competition. Because carrion is rich in nutrients but appears ephemerally in nature (i.e., timing and duration), it is advantageous for scavengers to first detect and locate it before other organisms arrive, resulting in resource competition and strong selection (Selva et al. 2019). Generally, because resource patches of carrion can often be small relative to the scavenger, they can be monopolized and defended to maximize resource ability to an individual (Beasley et al. 2015). In many cases, the resource is too large for a single scavenger to guard, resulting in scramble competition, which often occurs intraspecifically (as seen in hyenas; Tilson and Hamilton 1984). Finally, as the carrion resource becomes depleted, competition will intensify.

### Transfer of energy, nutrients, and diseases

Scavengers have the potential to relocate energy, nutrients, and contaminants within and between ecosystem boundaries (i.e., autochthonous and allochthonous, respectively; Richardson and Sato 2015). For example, aquatic insects have been documented to be important vectors for energy, nutrients, and contaminants in terrestrial food webs (Ballinger and Lake 2006, Jackson et al. 2021, Previšić et al. 2021). Indeed, freshwater ecosystems harbor high diversity and biomass of aquatic insects and most have an adult winged stage that can disperse out of aquatic ecosystems. Aquatic insects show important trophic plasticity (Fenoglio et al. 2014), and many lineages have been reported to feed on carrion including caddisfly larvae, beetles (larvae and adults), and midge larvae (Fenoglio et al. 2005, Walter et al. 2006, Fenoglio et al. 2010, Velasco and Millan 1998, Wartenberg et al. 2017, Dalal et al. 2020, Ebner et al. 2021). Midges can reach extremely high densities in some aquatic systems and are an important component of the diet of many terrestrial predators including spiders, lizards, birds, and bats (Baxter et al. 2005). Fully aquatic scavengers can also connect terrestrial and aquatic ecosystems. Crayfish and bullheads have both been shown to be effective scavengers on dead fish (Boros et al. 2020), and both taxa are commonly preyed on by birds and semiaquatic mammals.

Aquatic scavengers can also mediate the incorporation of terrestrial energy and materials into aquatic food webs. For instance, caddisfly larvae, which are an important component in the diet of aquatic species including fish (Morse et al. 2019), are known to feed on the carcasses of terrestrial vertebrates and invertebrates (Carlson et al. 2020; Lepori 2023). In another case, American alligators (*Alligator mississippiensis*) have been shown to consume the carrion of nesting birds, adding terrestrial subsidies to freshwater ecosystems (Gabel et al. 2019). In the Mara River of the Serengeti, aquatic scavengers including insects, fish, and crocodiles affect nutrient cycling and storage by feeding on the carcasses of wildebeest following mass drowning events during their annual migration. Following a mass (more than 100 wildebeest) drowning, 34% to 50% of the diet of fish was derived from wildebeest carcasses, either directly through scavenging or indirectly through feeding on scavenging invertebrates (Subalusky et al. 2017). The transfer of nutrients and energy from terrestrial ecosystems to freshwater via a freshwater scavenger has also been documented, highlighting their ability to connect ecosystems bidirectionally.

Finally, it is possible that scavengers also could affect the transmission of diseases and pathogens within freshwater ecosystems (discussed further below). First, pathogens rely on scavengers for inadvertent horizontal disease transmission from the infected material (e.g., carrion) to new hosts (VerCauteren et al. 2012). The study of wildlife diseases is challenging, and representation of freshwater scavengers as vectors is underrepresented in the literature. However, there is more evidence for freshwater scavengers to limit the spread of pathogens and diseases. Scavengers can reduce the number of hosts by consuming infected carcasses, therefore removing the source and decreasing transmission to new hosts (Rudolf and Antonovics 2007).

## Freshwater scavengers benefit people

Scavengers and humans have interacted for thousands of years, and although this relationship has evolved over time, we continue to benefit from the ecosystem services provided by this group of animals (Dominguez-Rodrigo and Pickering 2003, Lozano et al. 2019). The importance of scavengers is often overlooked by people, whereby scavengers have been persecuted for their negative impacts on livestock, property, and human life (Ogada et al. 2012). It is plausible that scavengers within freshwater ecosystems are even more neglected, because these ecosystems are so often out of sight, out of mind (Cooke et al. 2021). Freshwater scavengers play a crucial role within their ecosystems that results in myriad direct and indirect benefits for people across the four different types of ecosystem services: regulating (i.e., related to the regulation of ecosystems), supporting (i.e., support other ecosystem services), cultural (nonmaterial benefits), and provisioning (i.e., products obtained from the ecosystem; table 2; Moleon et al. 2014).

**Table 2.** Different types of ecosystem services provided by freshwater scavengers with examples.

Ecosystem service type	Freshwater scavenger context	Freshwater example	References
Regulating	Removal of animal debris Disease and pest control Improvement of water quality	Freshwater turtles improved water quality after simulated fish kills	Moleon et al. 2014, Le Sage et al. 2019, Santori et al. 2020
Supporting	Nutrient cycling via breakdown of organic matter Nutrient vectors across ecosystem types Contribute to overall biodiversity	Insects, fish, and crocodiles transfer nutrients from wildebeest carcasses to freshwater ecosystems	Payne and Moore 2006, Subalusky et al. 2017, Anguilera-Alcala et al. 2020
Cultural	Provide spiritual, religious, inspirational, and aesthetic value Provide recreational and ecotourism opportunities	Freshwater turtles have played an important role to Indigenous peoples as rattles and percussion instruments	Jackson and Levine 2002, Anguilera-Alcala et al. 2020
Provisioning	Harvesting of scavengers Increased agricultural output via pest consumption of scavengers	Harvest and consumption of crayfish in the southern United States	Alford et al. 2017, Teng et al. 2016

First, freshwater scavengers contribute to regulating services through disease and pest control and through the removal of animal debris (Moleon et al. 2014). As freshwater scavengers consume host species, they reduce the risk of disease transmission, which benefits both people and other freshwater animals (O'Bryan et al. 2018). For instance, freshwater scavenging invertebrates resulted in decreased transmission of Ranavirus among long-toed salamander larvae (*Ambystoma macrodactylum*; Le Sage et al. 2019). In addition, the removal of animal debris by freshwater scavengers has played a hygienic role, whereby carcasses can result in hypoxia, algal blooms, and increased ammonia and nitrates within freshwater ecosystems (Sargent and Galat 2002). In a mesocosm experiment, freshwater turtles contributed to regulating ecosystem services by improving water quality during simulated fish kills (Santori et al. 2020). Next, freshwater scavengers play a role in supporting ecosystem services through nutrient cycling and adding to biodiversity, which contribute to the well-being of society (Anguilera-Alcala et al. 2020). Freshwater scavengers also contribute to nutrient cycling in aquatic ecosystems, where they break down organic matter, releasing nutrients back to the environment (Boros et al. 2020). Furthermore, these animals can also act as nutrient vectors, transferring nutrients between systems (e.g., from lentic to terrestrial systems; figure 1e; Payne and Moore 2006), therefore redistributing nutrients. Third, despite their poor public reputation, freshwater scavengers contribute to important cultural ecosystem services by providing spiritual, religious, inspirational, and aesthetic values, as well as recreational and ecotourism opportunities (Anguilera-Alcala et al. 2020). For example, freshwater turtles have been important for Indigenous peoples across North America for millennia, where the carapaces are used as rattles and percussion instruments, therefore representing an important cultural symbol (figure 1d; e.g., Jackson and Levine 2002). Finally, freshwater scavengers provide provisioning ecosystem services in the form of direct harvesting and contributing to agriculture. For example, freshwater crayfish (e.g., *Procambarus clarkii*) are harvested from wetlands for human consumption throughout the southern United States (Alford et al. 2017). Although we have explored each of the four types of ecosystem services in the present article, freshwater scavengers require substantially more research to understand the full benefits of these important animals.

## Scavengers in the Anthropocene

Widespread declines of freshwater biodiversity (see Harrison et al. 2018) will undoubtedly affect freshwater scavengers and the ecosystem services that they provide. Although there has been limited research on how loss of freshwater biodiversity may affect scavengers, we have collectively identified four main ways in which freshwater scavengers may be anticipated to be affected by anthropogenic change: direct effects on their populations affecting their ability to carry out their functional role in ecosystems, direct effects on the availability or quality of their forage base (which may be a result of shifts in the distribution of scavengers and forage), direct effects of bioaccumulation of pollutants and chemicals, and indirect effects on their behavior and their distribution due to human activity.

First, direct effects on scavenger species are already observable as several key scavenging taxa are considered to be highly visible victims of global environmental change, including crayfishes and freshwater turtles (figure 1a and 1d; Lovich et al. 2018). With the ongoing highly pathogenic H5 avian influenza global epizootic, many aquatic bird species such as gulls and anatids may face steep declines (Artois et al. 2009). To date, highly pathogenic avian influenza has caused spillover into avian and mammalian predators and scavengers of affected aquatic birds resulting in mortality of bears, mink, raccoons, otters, and raptors. Insect populations are also declining in freshwater (Jähnig et al. 2021), affecting scavenging species such as beetles. Crayfish populations around the world have been affected by invasions of nonnative competitors, as well as transmission of communicable diseases such as crayfish plague (e.g., Bozzuto et al. 2024). From our knowledge of terrestrial systems, the loss of dominant scavengers (e.g., racoons, *Procyon lotor*) may not necessarily alter the scavenging community; however, the efficiency of carrion removal may decrease, meaning that more carcasses go untouched and decomposition will proceed more slowly (Olson et al. 2012). As ecosystems are forced to respond to increasing uncertainty of events such as floods, droughts, anoxia, thermal extremes, and extreme weather, episodic mortality events (e.g., fish kills) will require scavengers to quickly recycle biomass to regulate water quality. Forage provision through mortality inputs may become more unpredictable. Santori and colleagues (2020) demonstrated how losses of these species is likely to negatively affect the resilience of systems afflicted by such pressures.

Second, freshwater scavengers are losing access to some species in their ecosystems as freshwater species diversity and abundance indices continue to spiral downward. A modeling exercise revealed that global biodiversity loss and redistribution has likely massively affected the availability of carcasses in freshwater, with implications for nutrient and matter availability to scavengers (Wenger et al. 2019). Moreover, significant losses of carnivores such as bears, otters, and eagles can be expected to negatively influence the availability of carrion to scavengers if fewer animals are being killed by predators (Levi et al. 2020). Where native species are lost or imperiled and nonnative species have moved in, scavengers will not necessarily be as negatively affected as predators that may fail to learn to exploit novel species. Indeed, there is no evidence that nonnative species, such as carp (*Cyprinus carpio*) in Spain, pink salmon (*Oncorhynchus gorbuscha*) in Norway, and nonnative bivalves in choice experiments are avoided by scavengers in favor of native counterparts (Dunlop et al. 2021, Orihuela‐Torres et al. 2022, Sanders and Mills 2022). In another example, the presence of an invasive scavenger, red swamp crayfish, in a Spanish river formed the bulk of the diet of native Eurasrion otter (Dettori et al. 2021). This means that shifting patterns of biodiversity may not have as negative impacts on scavengers as overall biodiversity losses. However, replacement of species with smaller counterparts or overall shifting size distributions of animals to cope with warming (Gardner et al. 2011) may reduce the number of animals that are able to benefit from a single carcass.

Third, scavengers play a critical role in mediating the bioaccumulation and bioamplification of pollutants within ecosystems, creating complex interactions between carrion, scavengers, and predators. Bioaccumulation is particularly concerning for scavenger species that may be long-lived with high biomass, including many species of freshwater turtles, which could influence the distribution and persistence of pollutants within these ecosystems (Iverson 1982, Congdon et al. 1986). For instance, pond sliders (*Trachemys scripta*) in a nuclear reactor reservoir in South Carolina derived over half of their diet from fish carrion, demonstrating their role in nutrient and pollutant cycling (Parmenter 1980). Moreover, increased toxicity of carrion because of bioamplification of contaminants should be a concern, and Beale and colleagues (2022) found that Australian freshwater turtles were burdened with high levels of perfluoroalkylated substances as a result of consuming dead animals that had been exposed to these artificial compounds. Even smaller freshwater scavengers that have relatively short lifespans have the potential to have food-web-wide implications through bioamplification. For example, leeches feeding on fish carcasses in boreal lakes can accumulate 16% of the methyl mercury found in the carcasses, making them vectors of pollutant transfer to both aquatic and terrestrial predators (Cywinska and Davies 1989, Sarica et al. 2005). This demonstrates how scavengers can facilitate the redistribution of pollutants across trophic levels and ecosystem boundaries. In some cases, scavenging activity can even create a positive feedback loop of bioaccumulation. Laboratory experiments have shown that largemouth bass feeding on crayfish, which themselves consumed bass carrion, accumulated more methyl mercury than those fed methyl mercury–supplemented artificial diets. This highlights the potential for scavenger-mediated pathways to amplify pollutant concentrations within food webs (Bowling et al. 2011). By integrating these case studies, a clearer picture emerges of how scavengers mediate the movement of pollutants and nutrients, linking scavenging behavior to broader ecological and environmental processes.

Finally, where scavenger populations remain robust with access to carrion, there may be key indirect effects of the Anthropocene on their ability to carry out their role. Behavior is key to scavenging and encounter rates between animals and carrion govern the pace at which scavengers convert dead matter to energy. Gutiérrez‐Cánovas and colleagues (2020) demonstrated that scavengers with large home ranges drive higher efficiency carrion removal; large home ranges are typically associated with larger animals and older individuals within species, which requires targeted conservation efforts to maintain the longevity of species such as turtles and alligators that are long-lived and have the potential to be superscavengers within a population (e.g., Piczak et al. 2019a). However, urban areas can truncate home ranges for animals that avoid humans, and Etherington and colleagues (2023) revealed impacts of proximity to urban environments as a predictor of whether a carcass would be scavenged in a freshwater ecosystem. This would also apply to other kinds of human-mediated habitat loss (e.g., forest harvest or agriculture). Another anthropogenically mediated factor that could affect the efficacy of scavengers includes the introduction of supplemental carcasses through activities such as stocking (e.g., of invasive species including trout; Unger and Hickman 2019; or common carp, *Cyprinus carpio* in lakes; Weber and Brown 2016), agriculture or hunting (e.g., cattle or ungulates enter aquatic ecosystems, respectively; see Sebastián‐González et al. 2019). Taken together, we anticipate that scavengers may be affected by these four processes; however, there is much to be learned about how the freshwater biodiversity crisis will affect scavenging, and additional research is needed to fill these paucities.

## Management implications of scavengers in the Anthropocene

The Anthropocene is on us, and with it comes manifold changes in the structure and function of ecosystems. As was outlined above, those changes have consequences for scavengers. Given persistent and emerging threats, there is a need to consider how to manage and conserve scavengers in the Anthropocene. Admittedly, such efforts have been rather scant to date, with more examples from the terrestrial realm (see Patterson et al. 2022) than the freshwater realm.

One potential form of intervention is the provision of supplemental carcasses to support scavengers or their ecological functions (Fielding et al. 2014). In freshwater systems, there are examples where carcasses have been introduced to compensate for the loss of natural salmon stocks as part of ecosystem restoration (Roni et al. 2002). These efforts have yielded mixed results but generally benefit scavengers (indirectly) while contributing to the mobilization of marine-derived nutrients into lotic and riparian zones, supporting plant growth and future allochthonous inputs (Wheeler and Kavanagh 2017, Compton et al. 2006). However, the effectiveness of such interventions may be context dependent. For instance, both streams and lentic ecosystems are often isolated within the landscape, meaning carcass additions may only provide localized benefits while imposing significant economic costs for managers. In lotic systems, carcasses can be easily dragged by currents, undergo rapid physical breakdown, and ultimately become a resource solely for microbial communities, limiting their broader ecological benefits. Further research is needed to quantify the fate of these added carcasses and examine which taxa—spanning aquatic and terrestrial scavengers—are using these additional inputs. Moreover, the success of these interventions assumes that scavengers remain present in ecosystems where carcasses have historically declined, because their presence is vital to facilitating nutrient transfer. There are analogous examples of supplemental food provisions for highly endangered avian scavengers, such as vultures, which are often species-specific interventions aimed at population recovery (Cortés‐Avizanda et al. 2016). Therefore, although carcass addition can serve as a textbook example of ecosystem restoration, its limitations and broader implications must be critically evaluated to ensure its ecological and economic viability.

In the context of climate change, there may be a need for specific management actions targeted toward scavengers. Much research has focused on how to protect habitat for fish and other aquatic organisms that may become carrion. For instance, retaining vegetation that provides shade can maintain cooler water with less suspended sediment and is one objective that forest management can address. Conservation of riparian area shading along streams and forested wetlands can reduce the rate of heating that compromise survival of species, such as Pacific salmon and others (Martin et al. 2021). The protection of riparian vegetation also reduces sediment supplies from the banks and can absorb some excess nutrients. This protection should occur along small streams, because once water is heated that accumulated energy is hard to dissipate (Blandon et al. 2018). This is particularly true as climate change leads to warmer, drier summers in some places, making lower volume surface waters more susceptible to heat. Likewise, restoration of disturbed riparian areas can go a long way toward meeting many objectives. Streamside protection may also help reduce flood damage from the increasingly severe storms associated with climate change. At a larger scale, watershed management to minimize changes to natural hydrological regimes would likely benefit the carrion–scavenger interaction, and other ecological processes. In addition, protecting habitat for scavengers and sources of carrion helps sustain the ecological process. Moreover, given the role that scavengers play in helping to clean up biological events (such as fish kills; for examples involving turtles, see Santori et al. 2020) and the apparent increase in such events moderated by climate change, there may be need to actively manage scavenger populations to ensure that ecosystem services can be provided as needed. Much of climate change research and management focuses on the top and bottom of food chains, whereas scavengers play important but often overlooked roles. Because ecosystems experience flux attributed to both climate change and the introduction of invasive species, fostering redundancy in ecological roles and enhancing resilience requires considering ecosystem structure from a functional perspective.

A wide range of management actions can be implemented to sustain and enhance scavenger–carrion interactions, which are crucial for maintaining ecosystem health and biodiversity. One fundamental approach is the removal of barriers to the movement of both scavengers and their carrion resources. These barriers—whether physical, such as dams or fences, or ecological, such as habitat fragmentation—can limit the ability of aquatic scavengers to access essential food sources and disrupt vital ecological processes (see Patterson et al. 2023). Ensuring that scavengers can move freely across their habitats is essential for maintaining healthy populations. Another important strategy is the removal or control of invasive species that may alter habitats or directly compete with scavengers or their carrion resources (Ricciardi and MacIsaac 2011). Invasive species often outcompete native species for food or space, which can lead to a decrease in scavenger populations and the availability of carrion. Effective control or eradication of these invaders can help restore ecological balance and support the persistence of scavenger–carrion interactions. The management of contaminants and excess nutrients in aquatic environments is also critical. Pollutants, such as heavy metals, plastics, and chemical runoff, can impair the health of species involved in scavenger–carrion interactions, including both the scavengers themselves and the organisms that provide carrion (e.g., Sultana et al. 2021). In addition, in cases where certain species have been lost or their populations diminished, translocating native species may be a viable option. This could involve relocating individuals from healthy populations to areas where scavenger populations have declined because of habitat loss, resource depletion, or other threats. By augmenting these populations, it may be possible to restore the ecological roles of scavengers and maintain the health of the ecosystems they support; however, this approach may be restricted to less mobile taxa and associated with economic costs.

Some scavengers are highly mobile such that they may cross jurisdictional boundaries. In freshwater systems, this is most pertinent in scavenging birds and migratory or otherwise transient fishes in large river systems (e.g., Arrondo et al. 2018). Ensuring that scavengers are considered in transboundary (could be within a nation or across national boundaries) conservation policies is necessary (Lambertucci et al. 2014). However, given that most transboundary policies focus on protecting exploited commercially valuable animals, many scavengers may suffer from a policy vacuum. From the highly mobile scavenging fish such as the goonch catfish (*Bagarius yarrelli*) in southeast Asia to scavenging birds that fly with ease across boundaries (e.g., turkey vultures; *Cathartes aura*), without coordinated policy protection and management, there is potential for scavenger species to be forgotten.

In many ways, one of the most important conservation actions for scavengers is to tell their stories to increase public awareness and ensure that they are not neglected or forgotten in policy and management (e.g., Ng et al. 2015). This is particularly salient in freshwater systems where the scavengers may be cryptic and underappreciated. Indeed, the premise of this article is to raise the profile of scavengers in freshwater including in the context of their bespoke management and conservation.

## Knowledge gaps and research needs

The role of scavengers in freshwater ecosystems remains significantly underresearched, leaving critical knowledge gaps that hinder a full understanding of their ecological importance. To guide future research effectively, it is essential to identify and prioritize key questions. One of the most fundamental gaps is understanding the extent to which facultative scavenging occurs in freshwater systems (Orihuela‐Torres et al. 2024). Although obligate scavengers are relatively well documented, much less is known about species that engage opportunistically in scavenging. Understanding the scope and ecological significance of facultative scavengers is essential for capturing the full range of scavenger activity in freshwater environments. Another area in need of further investigation is the spatial and temporal dynamics of scavenger behavior (e.g., Preiszner et al. 2024). Freshwater scavengers likely vary in their activity across different environmental conditions, carrion availability, and seasonal patterns. Research is needed to determine how these factors influence scavenger distribution and behavior over time and space. Such knowledge is vital for linking scavenger populations to broader ecosystem processes, such as nutrient cycling and food web dynamics. Human impacts, including land-use changes, habitat degradation, and pollution, also likely affect scavenger dynamics, but there is limited research on how these activities alter both scavenger populations and the availability of carrion. For example, anthropogenic activities resulting in habitat fragmentation can alter the ability of scavengers to move within their aquatic environments (e.g., Piczak et al. 2019b); however, the extent to which these barriers prevent scavenging remains unknown. More research is needed to understand how human activities interact with scavenger behaviors and to predict the potential consequences of these disruptions on freshwater ecosystems. The impact of invasive species on freshwater scavenger dynamics represents another critical knowledge gap. It is unclear whether native scavengers interact with nonnative carcasses and how invasive scavengers may alter ecological processes by competing with native species or altering scavenger–carrion interactions (Orihuela‐Torres et al. 2022). Understanding these interactions is essential for assessing the broader ecological impacts of invasive species on freshwater ecosystems. Climate change adds another layer of complexity, because it is expected to affect carrion availability, scavenger behavior, and ecosystem conditions. Shifts in temperature, extreme weather events, and changes in precipitation patterns may influence the timing and quality of carrion resources, as well as scavenger population dynamics. Although this has been studied in terrestrial systems (Marneweck et al. 2021), additional research is needed to understand how climate-induced changes will affect scavenger roles and their interactions with freshwater ecosystems. To address these gaps, empirical experimental studies are needed to explore scavenger-mediated processes across a range of freshwater habitats and species. By addressing these key research questions, future studies can significantly advance our understanding of the role of scavengers in freshwater ecosystems and help fill critical gaps in the literature.

## Conclusions

Obligate and facultative scavengers are common, albeit cryptic, in freshwater and riparian systems. We identified and synthesized diverse ways in which these animals benefit people and contribute to ecosystem structure and function. However, the world is changing, and we are currently facing a freshwater biodiversity crisis in the Anthropocene (Harrison et al. 2018). The extent to which threats facing freshwater biodiversity also affect scavengers is unclear, but failure to include scavengers in efforts to protect, restore, and manage freshwater systems would result in outcomes that could be far reaching and lead to extirpation of these organisms and potentially the alteration of entire ecosystems via direct and indirect processes. Ensuring that scavengers are included in bespoke management plans (especially related to protection policies for transboundary populations or species) as well as efforts to develop coordinated ecosystem management plans that explicitly include scavengers would represent major steps forward. Effectively communicating and celebrating the important role of freshwater and riparian scavengers is crucial for their integration into ecosystem management and for generating the public and political will needed to ensure their protection and sustainable management.

## Author Biography

Morgan L. Piczak (morganpiczak@gmail.com), Grégory Bulté, and Steven J. Cooke are affiliated with the Department of Biology and the Institute of Environmental and Interdisciplinary Science at Carleton University, in Ottawa, Ontario, Canada. Morgan L. Piczak, Robert J. Lennox are affiliated with the Department of Biology at Dalhousie University, in Halifax, Nova Scotia, Canada. Knut W. Vollset is affiliated with the NORCE Norwegian Research Centre, Laboratory for Freshwater Ecology and Inland Fisheries, in Bergen, Norway. Bálint Preiszner and Tibor Erős are affiliated with the HUN-REN Balaton Limnological Research Institute, Fish and Conservation Ecology Research Group, in Tihany, Hungary. Matt G. Keevil is affiliated with the School of Natural Sciences at Laurentian University, in Sudbury, Ontario, Canada. John S. Richardson is affiliated with the Department of Forest and Conservation Sciences at the University of British Columbia, in Vancouver, British Columbia, Canada.

## References

[bib1] Aguilera-Alcalá N, Morales-Reyes Z, Martín-López B, Moleón M, Sánchez-Zapata JA. 2020. Role of scavengers in providing non-material contributions to people. Ecological Indicators 117: 106643.

[bib2] Albert JS, Destouni G, Duke-Sylvester SM, Magurran AE, Oberdorff T, Reis RE, Ripple WJ. 2021. Scientists’ warning to humanity on the freshwater biodiversity crisis. Ambio 50: 85–94. 32040746 10.1007/s13280-020-01318-8 PMC7708569

[bib3] Alford AB, Kaminski RM, Grado SC, D'Abramo LR, Avery JL. 2017. Harvest of crayfish as an ecosystem service of wetlands compared to production systems with planted forage. Aquaculture Economics and Management 21: 295–313.

[bib6] Arrondo E, Moleón M, Cortés-Avizanda A, Jiménez J, Beja P, Sánchez-Zapata JA, Donázar JA. 2018. Invisible barriers: Differential sanitary regulations constrain vulture movements across country borders. Biological Conservation 219: 46–52.

[bib7] Artois M, Bicout D, Doctrinal D, Fouchier R, Gavier-Widen D, Globig A, Olsen B. 2009. Outbreaks of highly pathogenic avian influenza in Europe: The risks associated with wild birds. Revue Scientifique et Technique de l'Oie 28: 69. 10.20506/rst.28.1.1854 19618620

[bib8] Bailey CJ, Moore JW. 2020. Resource pulses increase the diversity of successful competitors in a multi-species stream fish assemblage. Ecosphere 11: e03211.

[bib9] Ballinger A, Lake PS. 2006. Energy and nutrient fluxes from rivers and streams into terrestrial food webs. Marine and Freshwater Research 57: 15–28.

[bib10] Barton PS, Cunningham SA, Lindenmayer DB, Manning AD. 2013. The role of carrion in maintaining biodiversity and ecological processes in terrestrial ecosystems. Oecologia 171: 761–772. 23007807 10.1007/s00442-012-2460-3

[bib11] Baxter CV, Fausch KD, Carl Saunders W. 2005. Tangled webs: Reciprocal flows of invertebrate prey link streams and riparian zones. Freshwater Biology 50: 201–220.

[bib12] Beale DJ, Hillyer K, Nilsson S, Limpus D, Bose U, Broadbent JA, Vardy S. 2022. Bioaccumulation and metabolic response of PFAS mixtures in wild-caught freshwater turtles (*Emydura macquarii macquarii*) using omics-based ecosurveillance techniques. Science of the Total Environment 806: 151264. 34715216 10.1016/j.scitotenv.2021.151264

[bib13] Beasley JC, Olson ZH, DeVault TL. 2015. Ecological role of vertebrate scavengers. Pages 107–127 in Benbow ME, Tomberlin JK, Tarone AM eds. Carrion Ecology, Evolution, and Their Applications. CRC Press.

[bib14] Benbow ME, Tomberlin JK, Tarone AM, eds. 2015. Carrion Ecology, Evolution, and Their Applications. CRC Press.

[bib15] Benbow ME, Receveur JP, Lamberti GA. 2020. Death and decomposition in aquatic ecosystems. Frontiers in Ecology and Evolution 8: 17.

[bib16] Bladon KD, Sebura C, Cook NA, Bywater-Reyes S, Reiter M. 2018. A multicatchment analysis of headwater and downstream temperature effects from contemporary forest harvesting. Hydrological Processes 32: 293–304.

[bib17] Boros G, Czeglédi I, Erős T, Preiszner B 2020. Scavenger-driven fish carcass decomposition and phosphorus recycling: Laboratory experiments with freshwater fish and crayfish. Freshwater Biology 65: 1740–1751.

[bib18] Bowling AM, Hammerschmidt CR, Oris JT. 2011. Necrophagy by a benthic omnivore influences biomagnification of methylmercury in fish. Aquatic Toxicology 102: 134–141. 21356175 10.1016/j.aquatox.2011.01.006

[bib19] Bozzuto C, Schmidt-Posthaus H, Adrian-Kalchhauser I, Pisano SRR. 2024. Towards an eco-epidemiological framework for managing freshwater crayfish communities confronted with crayfish plague. Journal of Freshwater Ecology 39: 2405722.

[bib20] Britton JC, Morton B. 1994. Marine carrion and scavengers. Oceanography and Marine Biology: An Annual Review 32: 369–434.

[bib21] Brown CR, Sergio AJ, Bate CS, Koopman N, Roland JB, Notman-Grobler OD, Lennox RJ. 2024. A review of migratory *Alosidae* marine ecology in the northwest Atlantic. Journal of Fish Biology 106: 15977. 10.1111/jfb.15977 PMC11949747 39523025

[bib22] Butterworth NJ, Benbow ME, Barton PS. 2023. The ephemeral resource patch concept. Biological Reviews 98: 697–726. 36517934 10.1111/brv.12926

[bib23] Carlson SM, Rodriguez-Lozano P, Moidu H, Leidy RA. 2020. Scavenging of animal carcasses by *Gumaga nigricula* (Sericostomatidae, Trichoptera), an apparent herbivore. Western North American Naturalist 80: 551–555.

[bib24] Chaloner DT, Wipfli MS. 2002. Influence of decomposing Pacific salmon carcasses on macroinvertebrate growth and standing stock in southeastern Alaska streams. Journal of the North American Benthological Society 21: 430–442.

[bib25] Chidami S, Amyot M. 2008. Fish decomposition in boreal lakes and biogeochemical implications. Limnology and Oceanography 53: 1988–1996.

[bib26] Clua E, Chauvet C, Read T, Werry JM, Lee SY. 2013. Behavioural patterns of a tiger shark (*Galeocerdo cuvier*) feeding aggregation at a blue whale carcass in Prony Bay, New Caledonia. Marine and Freshwater Behaviour and Physiology 46: 1–20.

[bib27] Collins MA, Bailey DM, Ruxton GD, Priede IG. 2005. Trends in body size across an environmental gradient: A differential response in scavenging and non-scavenging demersal deep-sea fish. Proceedings of the Royal Society B 272: 2051–2057. 16191616 10.1098/rspb.2005.3189 PMC1559896

[bib29a] Compton JE et al. 2006. Ecological and water quality consequences of nutrient addition for salmon restoration in the Pacific Northwest. Frontiers in Ecology and the Environment 4: 18–26.

[bib29] Congdon JD, Greene JL, Gibbons JW. 1986. Biomass of freshwater turtles: A geographic comparison. American Midland Naturalist 115: 165–173.

[bib30] Cooke SJ, Lynch AJ, Piccolo JJ, Olden JD, Reid AJ, Ormerod SJ. 2021. Stewardship and management of freshwater ecosystems: From Leopold's land ethic to a freshwater ethic. Aquatic Conservation: Marine and Freshwater Ecosystems 31: 1499–1511.

[bib31] Cortés-Avizanda A, Blanco G, DeVault TL, Markandya A, Virani MZ, Brandt J, Donázar JA. 2016. Supplementary feeding and endangered avian scavengers: Benefits, caveats, and controversies. Frontiers in Ecology and the Environment 14: 191–199.

[bib32] Cywinska A, Davies RW. 1989. Predation on the erpobdellid leech *Nephelopsis obscura* in the laboratory. Canadian Journal of Zoology 67: 2689–2693.

[bib33] Dalal J, Sharma S, Bhardwaj T, Dhattarwal SK, Verma K. 2020. Seasonal study of the decomposition pattern and insects on a submerged pig cadaver. Journal of Forensic and Legal Medicine 74: 102023. 32784108 10.1016/j.jflm.2020.102023

[bib34] Dettori EE, Balestrieri A, Zapata-Perez VM, Bruno D, Rubio-Saura N, Robledano-Aymerich F. 2021. Distribution and diet of recovering Eurasian otter (*Lutra lutra*) along the natural-to-urban habitat gradient (river Segura, SE Spain). Urban Ecosystems 24: 1221–1230.

[bib35] DeVault TL, OE R Jr, Shivik JA. 2003. Scavenging by vertebrates: Behavioral, ecological, and evolutionary perspectives on an important energy transfer pathway in terrestrial ecosystems. Oikos 102: 225–234.

[bib36] Di Marco M, Chapman S, Althor G, Kearney S, Besancon C, Butt N, Watson JE. 2017. Changing trends and persisting biases in three decades of conservation science. Global Ecology and Conservation 10: 32–42.

[bib37] Domínguez-Rodrigo M, Pickering TR. 2003. Early hominid hunting and scavenging: A zooarcheological review. Evolutionary Anthropology: Issues, News, and Reviews 12: 275–282.

[bib38] Dunlop KM, Wipfli M, Muladal R, Wierzbinski G. 2021. Terrestrial and semi-aquatic scavengers on invasive Pacific pink salmon (*Oncorhynchus gorbuscha*) carcasses in a riparian ecosystem in northern Norway. Biological Invasions 23: 973–979.

[bib39] Ebner BC, Donaldson JA, Marshall J, Starrs D, Freeman AB. 2021. Diving beetles strip eel to the bone. Food Webs 27: e00188.

[bib40] Etherington BS, Piczak ML, LaRochelle L, Gallagher AJ, Cooke SJ. 2023. Effects of anthropogenic activities on scavenger communities in freshwater riparian zones of eastern Ontario. Aquatic Ecology 57: 115–125.

[bib41] Fenoglio S, Bo T, Agosta P, Cucco M. 2005. Mass loss and macroinvertebrate colonisation of fish carcasses in riffles and pools of a NW Italian stream. Hydrobiologia 532: 111–122.

[bib42] Fenoglio S, Bo T, Cammarata M, Malacarne G, Del Frate G. 2010. Contribution of macro- and micro-consumers to the decomposition of fish carcasses in low-order streams: An experimental study. Hydrobiologia 637: 219–228

[bib44] Fenoglio S, Merritt RW, Cummins KW. 2014. Why do no specialized necrophagous species exist among aquatic insects? Freshwater Science 33: 711–715.

[bib45] Fielding D, Newey S, van der Wal R, Irvine RJ. 2014. Carcass provisioning to support scavengers: Evaluating a controversial nature conservation practice. Ambio 43: 810–819. 24366570 10.1007/s13280-013-0469-4 PMC4165841

[bib47] Gabel W, Frederick P, Zabala J. 2019. Nestling carcasses from colonially breeding wading birds: Patterns of access and energetic relevance for a vertebrate scavenger community. Scientific Reports 9: 14512. 31601853 10.1038/s41598-019-50986-4 PMC6787207

[bib48] Gardner JL, Peters A, Kearney MR, Joseph L, Heinsohn R. 2011. Declining body size: A third universal response to warming?. Trends in Ecology and Evolution 26: 285–291. 21470708 10.1016/j.tree.2011.03.005

[bib49] Gutiérrez-Cánovas C, Moleón M, Mateo-Tomás P, Olea PP, Sebastián-González E, Sánchez-Zapata JA. 2020. Large home range scavengers support higher rates of carcass removal. Functional Ecology 34: 1921–1932.

[bib50] Harrison I, Abell R, Darwall W, Thieme ML, Tickner D, Timboe I. 2018. The freshwater biodiversity crisis. Science 362: 1369–1369. 10.1126/science.aav9242 30573621

[bib52] Heintz R, Wipfli MS, Hudson JP. 2010. Identification of marine-derived lipids in Juvenile Coho Salmon and Aquatic Insects through Fatty Acid Analysis. Transactions of the American Fisheries Society 139: 840–854.

[bib53] Holt RD. 2008. Theoretical perspectives on resource pulses. Ecology 89: 671–681. 18459331 10.1890/07-0348.1

[bib54] Hyndes GA, Berdan EL, Duarte C, Dugan JE, Emery KA, Hambäck PA, Schlacher TA. 2022. The role of inputs of marine wrack and carrion in sandy-beach ecosystems: A global review. Biological Reviews 97: 2127–2161. 35950352 10.1111/brv.12886 PMC9804821

[bib55] Iverson JB. 1982. Biomass in turtle populations: A neglected subject. Oecologia 55: 69–76. 28309904 10.1007/BF00386720

[bib57] Jackson JB, Levine VL. 2002. Singing for garfish: Music and woodland communities in eastern Oklahoma. Ethnomusicology 46: 284–306

[bib56] Jackson AK, Eagles-Smith C, Robinson WD. 2021. Differential reliance on aquatic prey subsidies influences mercury exposure in riparian arachnids and songbirds. Ecology and Evolution 11: 7003–7017. 10.1002/ece3.7549 34141271 PMC8207155

[bib58] Jähnig SC, Baranov V, Altermatt F, Cranston P, Friedrichs-Manthey M, Geist J, Domisch S. 2021. Revisiting global trends in freshwater insect biodiversity. WIREs Water 8: e1506.

[bib59] Kane A, Healy K, Guillerme T, Ruxton GD, Jackson AL. 2017. A recipe for scavenging in vertebrates–the natural history of a behaviour. Ecography 40: 324–334.

[bib60] Kiffney PM, Naman SM, Cram JM, Liermann M, Burrows DG. 2018. Multiple pathways of C and N incorporation by consumers across an experimental gradient of salmon carcasses. Ecosphere 9: e02197. 10.1002/ecs2.2197

[bib61] Kohler AE, Rugenski A, Taki D. 2008. Stream food web response to a salmon carcass analogue addition in two central Idaho, U.S.A. streams. Freshwater Biology 53: 446–460.

[bib62] Kurasawa A, Onishi Y, Koba K, Fukushima K, Uno H. 2023. Multipath ecological influence of an iteroparous fish migration from Lake Biwa to an alluvial stream. Freshwater Biology 68: 1400–1412.

[bib63] Lambertucci SA, Alarcón PA, Hiraldo F, Sanchez-Zapata JA, Blanco G, Donázar JA. 2014. Apex scavenger movements call for transboundary conservation policies. Biological Conservation 170: 145–150.

[bib64] Lepori F. 2023. Consumption of terrestrial invertebrates by limnephilid caddisflies (Trichoptera: Limnephilidae) indicate an overlooked link in stream-riparian food webs. Food Webs 34: e00266.

[bib66] Le Sage MJ, Towey BD, Brunner JL. 2019. Do scavengers prevent or promote disease transmission? The effect of invertebrate scavenging on Ranavirus transmission. Functional Ecology 33: 1342–1350.

[bib67] Levi T, Hilderbrand GV, Hocking MD, Quinn TP, White KS, Adams MS, Wilmers CC. 2020. Community ecology and conservation of bear-salmon ecosystems. Frontiers in Ecology and Evolution 8: 513304.

[bib68] Lisney TJ, Wagner HJ, Collin SP. 2018. Ontogenetic shifts in the number of axons in the olfactory tract and optic nerve in two species of deep-sea grenadier fish (Gadiformes: Macrouridae: Coryphaenoides). Frontiers in Ecology and Evolution 6: 168.

[bib69] Lovich JE, Ennen JR, Agha M, Whitfield Gibbons J. 2018. Where have all the turtles gone, and why does it matter? BioScience 68: 771–781. 10.1093/biosci/biy095,

[bib70] Lozano J, Olszańska A, Morales-Reyes Z, Castro AA, Malo AF, Moleón M, Martín-López B. 2019. Human-carnivore relations: A systematic review. Biological Conservation 237: 480–492.

[bib71] Marneweck CJ, Katzner TE, Jachowski DS. 2021. Predicted climate-induced reductions in scavenging in eastern North America. Global Change Biology 27: 3383–3394. 33894030 10.1111/gcb.15653

[bib73] Martin DJ, Kroll AJ, Knoth JL. 2021. An evidence-based review of the effectiveness of riparian buffers to maintain stream temperature and stream-associated amphibian populations in the Pacific Northwest of Canada and the United States. Forest Ecology and Management 491: 119190.

[bib74] Matsushima R. 2021. Evidence of morphological adaptation to life underwater: Sternal keel affects swimming speed in giant water scavenger beetles (Coleoptera: Hydrophilidae: Hydrophilini). Canadian Journal of Zoology 99: 363–367.

[bib75] Minakawa N, Gara RI, Honea JM. 2002. Increased individual growth rate and community biomass of stream insects associated with salmon carcasses. Journal of the North American Benthological Society 21: 651–659

[bib76] Moleón M, Sanchez-Zapata JA, Margalida A, Carrete M, Owen-Smith N, Donázar JA. 2014. Humans and scavengers: The evolution of interactions and ecosystem services. BioScience 64: 394–403.

[bib77] Moleón M, Selva N, Quaggiotto MM, Bailey DM, Cortés-Avizanda A, DeVault TL. 2019. Carrion availability in space and time. Pages 23–44 in Olea PP, Mateo-Tomás P, Sánchez-Zapata JA, eds. Carrion Ecology and Management. Springer Nature.

[bib78] Momot WT, Gowing H, Jones PD. 1978. The dynamics of crayfish and their role in ecosystems. American Midland Naturalist 99: 10–35.

[bib79] Morse JC, Frandsen PB, Graf W, Thomas JA. 2019. Diversity and ecosystem services of Trichoptera. Insects 10: 125. 31052441 10.3390/insects10050125 PMC6572163

[bib80] Ng DJ, McGowan PJ, Raghavan R, Cai Y, Cumberlidge N, Davison G, Yeo DCJ. 2015. Conservation Strategy for the Singapore Freshwater Crab Johora singaporensis. National Parks Singapore.

[bib81] Nowlin WH, González MJ, Vanni MJ, Stevens MHH, Fields MW, Valente JJ. 2007. Allochthonous subsidy of periodical cicadas affects the dynamics and stability of pond communities. Ecology 88: 2174–2186. 17918396 10.1890/06-0570.1

[bib82] O'Bryan CJ, Braczkowski AR, Beyer HL, Carter NH, Watson JE, McDonald-Madden E. 2018. The contribution of predators and scavengers to human well-being. Nature Ecology and Evolution 2: 229–236. 29348647 10.1038/s41559-017-0421-2

[bib83] Ogada DL, Keesing F, Virani MZ. 2012. Dropping dead: Causes and consequences of vulture population declines worldwide. Annals of the New York Academy of Sciences 1249: 57–71. 22175274 10.1111/j.1749-6632.2011.06293.x

[bib84] Olson ZH, Beasley JC, DeVault TL, Rhodes Jr OE. 2012. Scavenger community response to the removal of a dominant scavenger. Oikos 121: 77–84.

[bib86] Orihuela-Torres A, Pérez-García JM, Sánchez-Zapata JA, Botella F, Sebastián-González E. 2022. Scavenger guild and consumption patterns of an invasive alien fish species in a Mediterranean wetland. Ecology and Evolution 12: e9133. 35923937 10.1002/ece3.9133 PMC9339756

[bib85] Orihuela-Torres A, Morales-Reyes Z, Hermoso V, Picazo F, Fernández DS, Pérez-García JM, Botella F, Sánchez-Zapata JA, Sebastián-González E. 2024. Carrion ecology in inland aquatic ecosystems: A systematic review. Biological Reviews 99: 1425–1443. 38509722 10.1111/brv.13075

[bib88] Parmenter RR. 1980. Effects of food availability and water temperature on the feeding ecology of pond sliders (*Chrysemys s. scripta*). Copeia 1980: 503–514.

[bib87] Parmenter RR, Lamarra VA. 1991. Nutrient cycling in a freshwater marsh: The decomposition of fish and waterfowl carrion. Limnology and Oceanography 36: 976–987.

[bib89] Patterson JR, DeVault TL, Beasley JC. 2022. Integrating terrestrial scavenging ecology into contemporary wildlife conservation and management. Ecology and Evolution 12: e9122. 35866022 10.1002/ece3.9122 PMC9289120

[bib90] Patterson JR, Szabo N, Beasley JC. 2023. Effects of urbanization on the efficiency and composition of vertebrate scavengers. Food Webs 37: e00317.

[bib91] Payne LX, Moore, J W. 2006. Mobile scavengers create hotspots of freshwater productivity. Oikos 115: 69–80.

[bib92] Piczak ML, Chow-Fraser P. 2019. Assessment of critical habitat for common snapping turtles (*Chelydra serpentina*) in an urbanized coastal wetland. Urban Ecosystems 22: 525–537.

[bib93] Piczak ML, Markle CE, Chow-Fraser P. 2019b. Decades of road mortality cause severe decline in a common snapping turtle (*Chelydra serpentina*) population from an urbanized wetland. Chelonian Conservation and Biology 18: 231–240.

[bib94] Polačik M, Jurajda P, Blažek R, Janáč M. 2015. Carcass feeding as a cryptic foraging mode in round goby *Neogobius melanostomus*. Journal of Fish Biology 87: 194–199. 26010420 10.1111/jfb.12708

[bib95] Preiszner B, Czeglédi I, Boros G, Liker A, Kern B, Erős T. 2020. Scavenging behaviour and size-dependent carcass consumption of the black bullhead (*Ameiurus melas*). Journal of Fish Biology 97: 1113–1119. 32743806 10.1111/jfb.14482

[bib96] Preiszner B, Szolnoki A, Czeglédi I, Erős T. 2024. Effects of turbidity and habitat complexity on the foraging behavior of the black bullhead (*Ameiurus melas*). Inland Waters 14: 49–57.

[bib98] Previšić A, Vilenica M, Vučković N, Petrović M, Rožman M. 2021. Aquatic insects transfer pharmaceuticals and endocrine disruptors from aquatic to terrestrial ecosystems. Environmental Science and Technology 55: 3736–3746. 10.1021/acs.est.0c07609. 33650859 PMC8031366

[bib99] Ricciardi A, MacIsaac HJ. 2011. Impacts of biological invasions on freshwater ecosystems. Pages 211–224 in Richardson DM, ed. Fifty Years of Invasion Ecology: The Legacy of Charles Elton. Wiley.

[bib100] Richardson JS, Neill WE. 1991. Indirect effects of detritus manipulations in a montane stream. Canadian Journal of Fisheries and Aquatic Sciences 48: 776–783.

[bib101] Richardson JS, Sato T. 2015. Resource flows across freshwater-terrestrial boundaries and influence on processes linking adjacent ecosystems. Ecohydrology 8: 406–415.

[bib102a] Roni P, Beechie TJ, Bilby RE, Leonetti FE, Pollock MM, Pess GR. 2002. A review of stream restoration techniques and a hierarchical strategy for prioritizing restoration in Pacific Northwest watersheds. North American Journal of Fisheries Management 22: 1–20.

[bib102] Rudolf VH, Antonovics J. 2007. Disease transmission by cannibalism: Rare event or common occurrence?. Proceedings of the Royal Society B 274: 1205–1210. 17327205 10.1098/rspb.2006.0449 PMC2189571

[bib103] Samways KM, Blair TJ, Charest MA, Cunjak RA. 2017. Effects of spawning Atlantic salmon (Salmo salar) on total lipid content and fatty acid composition of river food webs. Ecosphere 8: e01818. doi:10.1002/ecs2.1818

[bib104] Sanders H, Mills DN. 2022. Predation preference of signal crayfish (*Pacifastacus leniusculus*) on native and invasive bivalve species. River Research and Applications 38: 1469–1480.

[bib105] Santori C, Spencer RJ, Thompson MB, Whittington CM, Burd TH, Currie SB., Finter TJ., Van Dyke JU. 2020. Scavenging by threatened turtles regulates freshwater ecosystem health during fish kills. Scientific Reports 10: 14383. 32943647 10.1038/s41598-020-71544-3 PMC7499268

[bib106] Sargent JC, Galat DL. 2002. Fish mortality and physicochemistry in a managed floodplain wetland. Wetlands Ecology and Management 10: 113–119.

[bib107] Sarica J, Amyot M, Hare L, Blanchfield P, Bodaly RD, Hintelmann H, Lucotte M. 2005. Mercury transfer from fish carcasses to scavengers in boreal lakes: The use of stable isotopes of mercury. Environmental Pollution 134: 13–22. 15572220 10.1016/j.envpol.2004.07.020

[bib109] Schindler DE, Scheuerell MD, Moore JW, Gende SM, Francis TB, Palen WJ. 2003. Pacific salmon and the ecology of coastal ecosystems. Frontiers in Ecology and the Environment 1: 31–37.

[bib108] Schindler DE, Armstrong JB, Bentley KT, Jankowski K, Lisi PJ, Payne LX. 2013. Riding the crimson tide: Mobile terrestrial consumers track phenological variation in spawning of an anadromous fish. Biology Letters 9: 20130048. 23554279 10.1098/rsbl.2013.0048 PMC3645035

[bib110] Schlacher TA, Strydom S, Connolly RM, Schoeman D. 2013. Donor-control of scavenging food webs at the land-ocean interface. PLOS ONE 8: e68221. 23826379 10.1371/journal.pone.0068221 PMC3694906

[bib111a] Sebastián-González E et al. 2019. Scavenging in the Anthropocene: Human impact drives vertebrate scavenger species richness at a global scale. Global Change Biology 25: 3005–3017. 10.1111/gcb.14708 31127672

[bib111] Selva N, Moleón M, Sebastián-González E, DeVault TL, Quaggiotto MM, Bailey DM., Margalida A. 2019. Vertebrate scavenging communities. Pages 71–99 in Olea PP, Mateo-Tomás P, Sánchez-Zapata JA, eds. Carrion Ecology and Management. Springer Nature.

[bib112] Stepanian PM, Entrekin SA, Wainwright CE, Mirkovic D, Tank JL, Kelly JF. 2020. Declines in an abundant aquatic insect, the burrowing mayfly, across major North American waterways. Proceedings of the National Academy of Sciences 117: 2987–2992. 10.1073/pnas.1913598117 PMC7022163 31964842

[bib114] Subalusky AL, Post DM. 2019. Context dependency of animal resource subsidies. Biological Reviews 94: 517–538. 30246443 10.1111/brv.12465

[bib113] Subalusky AL, Dutton CL, Rosi EJ, Post DM. 2017. Annual mass drownings of the Serengeti wildebeest migration influence nutrient cycling and storage in the Mara River. Proceedings of the National Academy of Sciences 114: 7647–7652. 10.1073/pnas.1614778114 PMC5530641 28630330

[bib115] Sultana S, Al-Ghanim KA, Khan QF, Al-Misned F, Atique U, Ahmed Z, Mahboob S. 2021. Comparative assessment of heavy metal bioaccumulation in skeletal muscles of softshell and hard-shell freshwater turtles. Journal of King Saud University-Science 33: 101463.

[bib116] Tamburri MN, Barry JP. 1999. Adaptations for scavenging by three diverse bathyla species, *Eptatretus stouti, Neptunea amianta*, and *Orchomene obtusus*. Deep Sea Research Part I: Oceanographic Research Papers 46: 2079–2093.

[bib117] Tilson RL , III 1984. Social dominance and feeding patterns of spotted hyaenas. Animal Behaviour 32: 715–724.

[bib118a] Unger S, Hickman C. 2019. Report on the short-term scavenging of decomposing native and non-native trout in Appalachian streams. Fishes 4: 17.

[bib118] Velasco J, Millan VH. 1998. Feeding habits of two large insects from a desert stream: *Abedus herberti* (Hemiptera: Belostomatidae) and *Thermonectus marmoratus* (Coleoptera: Dytiscidae). Aquatic Insects 20: 85–96.

[bib119] VerCauteren KC, Pilon JL, Nash PB, Phillips GE, Fischer JW. 2012. Prion remains infectious after passage through digestive system of American crows (*Corvus brachyrhynchos*). PLOS ONE 7: e45774 23082115 10.1371/journal.pone.0045774 PMC3474818

[bib120] Walter JK, Bilby RE, Fransen BR. 2006. Effects of Pacific salmon spawning and carcass availability on the caddisfly *Ecclisomyia conspersa* (Trichoptera: Limnephilidae). Freshwater Biology 51: 1211–1218.

[bib121] Walters AW, Barnes RT, Post DM. 2009. Anadromous alewives (*Alosa pseudoharengus*) contribute marine-derived nutrients to coastal stream food webs. Canadian Journal of Fisheries and Aquatic Sciences 66: 439–448.

[bib122] Wartenberg N, Reinhard S, Nollert A, Staniczek AH, Kupfer A. 2017. Caddisfly larvae (Trichoptera: Phryganeidae) as scavengers of carcasses of the common frog *Rana temporaria* (Amphibia: Ranidae). Salamandra 53: 458–460.

[bib123] Weber MJ, Brown ML. 2016. Effects of resource pulses on nutrient availability, ecosystem productivity, and temporal variability following a stochastic disturbance in eutrophic glacial lakes. Hydrobiologia 771: 165–177.

[bib124] Wenger SJ, Subalusky AL, Freeman MC. 2019. The missing dead: The lost role of animal remains in nutrient cycling in North American Rivers. Food Webs 18: e00106.

[bib125a] Wheeler TA, Kavanagh KL. 2017. Soil biogeochemical responses to the deposition of anadromous fish carcasses in inland riparian forests of the Pacific Northwest, USA. Canadian Journal of Forest Research 47: 1506–1516.

[bib125] Wilson RR, Smith KL. 1984. Effect of near-bottom currents on detection of bait by the abyssal grenadier fishes Coryphaenoides spp., recorded in situ with a video camera on a free vehicle. Marine Biology 84: 83–91.

[bib126] Yang LH, Bastow JL, Spence KO, Wright AN. 2008. What can we learn from resource pulses? Ecology 89: 621–634. 18459327 10.1890/07-0175.1

[bib127] Zhang YJN, Richardson JS, Kolodziejczyk R. 2003. Impacts of marine-derived nutrients on stream ecosystem functioning. Proceedings of the Royal Society of London B 270: 2117–2123. 10.1098/rspb.2003.2478 PMC1691481 14561274

